# Health of Buffalo

**Published:** 1846-09

**Authors:** 


*Health of Buffalo.*—The present summer has been characterized by continued, uniform, elevated temperature, and by less than the average quantity of rain. As respects the temperature, it has exceeded that of any season during the last ten years. There has, however, been less sickness than usually attends our summer months. Our most frequent disease, cholera infantum, or "summer complaint," has been very considerably less prevalent than in former years. Fruit has been quite as abundant as ever, and it is always brought here in great abundance. If we were to draw any deductions as to the *propter hoc*, we should say that the immunity from the complaints of the season has been owing to the constancy of the temperature. We suspect that to sudden alterations in this respect, more than to any other climatic influence, is due the occurrence of the summer diseases of children.

As respects the general salubrity of this locality, we believe it may fairly challenge competition with any other. The atmosphere is clear and pure, remarkably free from humidity, and, except some places in a small portion of the outskirts of the city, wholly devoid of miasmata. The heat of summer very rarely conflicts with comfort, and the nights are almost invariably refreshingly cool. Our water, notwithstanding a kind of traditionary idea of lime, which is kept alive by those who prefer some artificial improvement upon the native element, is equal to water any where; and we would not hesitate to pit that taken from Lake Erie against the famous "Croton," which the New-Yorkers think so delicious. Altogether, if a Buffalo summer be not entirely satisfactory, we must attribute it to an indisposition, or an inability to appreciate the gifts of Providence—but we are digressing. In speaking of the health of Buffalo, we should state that among the Lake Mariners intermittent and remittent fevers are particularly rife the present summer. This is not owing to their exposure to miasmatic influences here, but at Western ports where such influences abound to a greater or less degree. If we are correctly informed these fevers prevail on the borders of the Lakes west of us more the present summer, than for some years past. Continued drought is generally supposed to furnish the explanation of their increased prevalence at particular seasons, owing to larger portions of soil capable of giving off miasmatic emanations being left exposed, by evaporation of the water covering their surface. If this be correct it will apply in the present instance. Whether it be the true explanation or not, we have nothing better to propose, nor are we aware that any other has been suggested which is more plausible.

